# Cadonilimab plus chemotherapy as first-line treatment for persistent, recurrent, or metastatic cervical cancer: a cost-effectiveness analysis

**DOI:** 10.3389/fimmu.2025.1562875

**Published:** 2025-04-03

**Authors:** Zuojuan Xiang, Zhengxiong Li, Xiaojuan Chen, Yingzhou Fu

**Affiliations:** ^1^ Department of Pharmacy, The Affiliated Cancer Hospital of Xiangya School of Medicine, Central South University, Hunan Cancer Hospital, Changsha, China; ^2^ School of Medical Informatics and Engineering, Xuzhou Medical University, Xuzhou, China

**Keywords:** cost-effectiveness, cadonilimab, cervical cancer, first-line treatment, partitioned survival model

## Abstract

**Background:**

Immunotherapy has made significant advancements in cervical cancer (CC) treatment; however, its efficacy remains limited in programmed death ligand 1 (PD-L1)-negative patients. Cadonilimab, the first bispecific antibody targeting both programmed death 1 (PD-1) and cytotoxic T lymphocyte-associated antigen-4 (CTLA-4), demonstrated superior efficacy and manageable safety as a first-line treatment for persistent, recurrent, or metastatic CC (p/r/m CC) in the phase III COMPASSION-16 trial. Notably, it showed significant survival benefits in PD-L1-negative patients. This study aimed to evaluate its cost-effectiveness from the perspective of the Chinese healthcare system.

**Methods:**

A partitioned survival model was developed based on data derived from the COMPASSION-16 trial. The model utilized a 3-week cycle length and a 10-year time horizon. The primary outcomes included costs, quality-adjusted life-years (QALYs), incremental cost-effectiveness ratio (ICER), incremental net monetary benefit (INMB), and incremental net health benefit (INHB). Additionally, sensitivity analyses, scenario analyses, and subgroup analyses were performed.

**Results:**

The cadonilimab plus chemotherapy regimen provided an additional 0.61 QALYs compared to chemotherapy alone, at an incremental cost of $42,486.54. This yielded an ICER of $70,220.88/QALY, exceeding the willingness-to-pay threshold of $38,042/QALY. The corresponding INMB and INHB were -$19,469.55 and -0.51 QALYs, respectively. Consequently, cadonilimab plus chemotherapy was not deemed to be cost-effective. Sensitivity analyses showed that the results remained consistent when each parameter varied within the predetermined range, indicating the model’s robustness. Subgroup analyses demonstrated no significant positive correlation between economic outcomes and PD-L1 expression levels. Notably, in the subgroup of patients who did not receive bevacizumab, cadonilimab plus chemotherapy emerged as a cost-effective alternative.

**Conclusion:**

In China, cadonilimab plus chemotherapy is not considered cost-effective compared to standard chemotherapy as a first-line treatment for the general p/r/m CC population. However, it represents a cost-effective option for patients ineligible for bevacizumab therapy.

## 1 Introduction

Despite the potential of human papillomavirus vaccination and cervical cancer (CC) screening to significantly reduce the incidence and mortality of CC, the global disease burden remains substantial (1). In China, the incidence of CC in 2022 reached 13.83 per 100,000, accounting for approximately 150,700 new cases, ranking fifth among female malignancies and constituting 23% of the global total. The mortality rate stood at 4.54 per 100,000, corresponding to approximately 55,700 deaths, which ranked sixth in cancer-related fatalities and represented 16% of global CC mortality (2, 3). Insufficient CC screening coverage, particularly in rural areas, leads to a significant number of cases being diagnosed at advanced stages. Moreover, the incidence and mortality rates of CC in rural areas are higher than those in urban areas, with the central region showing even greater rates compared to the western and eastern regions (4). Patients with persistent, recurrent, or metastatic CC (p/r/m CC) face limited therapeutic options and a poor prognosis, with a 5-year overall survival (OS) rate below 20% (5, 6). The phase III GOG240 trial demonstrated that adding the anti-angiogenic agent bevacizumab to systemic chemotherapy extended the median OS from 13.3 to 16.8 months (7). This evidence established platinum-based chemotherapy with optional bevacizumab supplementation as the standard first-line treatment for p/r/m CC. The KEYNOTE-826 trial signified a breakthrough in cervical cancer immunotherapy, demonstrating that integrating the PD-1 inhibitor pembrolizumab into the standard treatment regimen resulted in a median OS of 26.4 months (8). The subsequent BEATcc trial, which excluded patients ineligible for bevacizumab, evaluated the PD-L1 inhibitor atezolizumab in combination with chemotherapy and bevacizumab, demonstrating a remarkable median OS of 32.1 months (9). However, the KEYNOTE-826 trial revealed no survival benefit in patients with PD-L1 CPS <1. While the BEATcc trial did not take into account PD-L1 status as a stratifying factor, it remains uncertain whether PD-L1 negative patients could benefit from this treatment.

Cadonilimab is the first bispecific antibody approved to target both PD-1 and Cytotoxic T lymphocyte antigen-4 (CTLA-4). Its tetravalent structure results in higher avidity within the tumor microenvironment, where the density of PD-1 and CTLA-4 is greater than in normal peripheral tissues (10, 11). The Fc-null design eliminates detrimental effects such as antibody-dependent cellular cytotoxicity and phagocytosis, thereby enhancing the safety profile (12). The phase III COMPASSION-16 trial investigated the efficacy of cadonilimab plus chemotherapy (with or without bevacizumab) as a first-line treatment for patients with p/r/m CC. This multicenter study enrolled participants across 59 clinical sites in China. The cadonilimab group demonstrated a significantly prolonged median progression-free survival (PFS) (12.7 vs. 8.1 months, hazard ratio [HR] 0.62, 95% confidence interval [CI] 0.49-0.80, p < 0.0001). Although median OS in the cadonilimab group had not been reached at the time of data cutoff, a significant trend of benefit was observed (HR 0.64, 95% CI 0.48-0.86, p = 0.0011), with a 24-month OS rate of 62.2%. Notably, patients with PD-L1 CPS < 1 also exhibited significant clinical benefit, with an HR of 0.73 for PFS and 0.77 for OS (13). These results suggest therapeutic potential even in PD-L1-negative populations.

Cadonilimab has been included in the 2025 Chinese Basic Medical Insurance Drug List following price negotiations, which led to a 69.83% reduction in its price. In contrast, the prices of pembrolizumab and atezolizumab remain high. Reimbursement for cadonilimab is limited to patients with p/r/m CC who have failed previous platinum-based chemotherapy. This inclusion is expected to substantially improve treatment accessibility and increase hospital formulary adoption. However, there is currently a scarcity of literature on the economic evidence surrounding cadonilimab. This study assessed the cost-effectiveness of cadonilimab as a first-line treatment for p/r/m CC from the perspective of the Chinese healthcare system, utilizing data from the COMPASSION-16 trial. Its goal was to inform evidence-based health policy decisions and optimize the allocation of therapeutic resources.

## 2 Method

This study adhered to the Consolidated Health Economic Evaluation Reporting Standards (CHEERS) guidelines (14), as outlined in **Supplementary Table 1**.

### 2.1 Patients and intervention

The target population characteristics were based on the COMPASSION-16 trial. Eligible participants were women aged 18 to 75 who had been diagnosed with p/r/m CC. They were unsuitable for curative surgery or concurrent chemoradiotherapy, possessed an Eastern Cooperative Oncology Group (ECOG) performance status of 0 or 1, had at least one measurable lesion per the Response Evaluation Criteria in Solid Tumors (RECIST) version 1.1, and had not received prior systemic treatment. This study used existing published data, eliminating the need for ethical approval as no actual patient recruitment was involved.

Patients were randomly assigned in a 1:1 ratio to receive either cadonilimab (10 mg/kg, day 1) or placebo plus chemotherapy, with or without bevacizumab (15 mg/kg, day 1), administered every 3 weeks for six cycles. The chemotherapy regimen included either cisplatin (50 mg/m², day 1) or carboplatin (area under the curve [AUC] 4-5, day 1) in combination with paclitaxel (175 mg/m², day 1). Subsequently, cadonilimab or placebo, with or without bevacizumab, was administered as maintenance therapy until disease progression, unacceptable toxicity, or for up to two years of treatment. The COMPASSION-16 trial data guided the proportions of carboplatin, cisplatin, and bevacizumab used in the treatment. **Supplementary Table 2** contained detailed information regarding this. The calculation of drug dosages was based on assumptions of a body surface area of 1.64 m^2^, a body weight of 70 kg, and a creatinine clearance of 70 mL/min for Chinese women, as shown in **Table 1** (15).

**Table 1:** Key input parameters.

Parameters	Baseline value	Minimum	Maximum	Distribution	Source
Log-logistic OS survival model					
Cadonilimab plus chemotherapy group	shape=1.65, scale=32.74	–	–	–	Model fitting
Chemotherapy group	shape=1.84, scale=22.57	–	–	–	Model fitting
Log-logistic PFS survival model					
Cadonilimab plus chemotherapy group	shape=1.90, scale=13.52	–	–	–	Model fitting
Chemotherapy group	shape=2.18, scale=9.44	–	–	–	Model fitting
Cost ($)					
Routine laboratory tests per cycle	35.94	28.75	43.13	Gamma	(19)
Coagulation per unit	9.13	7.30	10.96	Gamma	(19)
Thyroid per unit	20.58	16.46	24.70	Gamma	(19)
Contrast enhanced CT per unit	268.87	215.10	322.64	Gamma	(19)
Best supportive care per cycle	274.36	219.49	329.23	Gamma	(19)
Terminal care	685.90	548.72	823.08	Gamma	(19)
Intravenous injection per unit	2.82	2.26	3.38	Gamma	(20)
Anti-tumor drug dispensing per unit	4.07	3.26	4.88	Gamma	(20)
Cost of drugs					
Cadonilimab/100mg	211.16	168.93	253.39	Gamma	Local charge
Cisplatin/100mg	13.18	9.04	21.57	Gamma	Local charge
Carboplatin/100mg	13.29	7.32	22.42	Gamma	Local charge
Paclitaxel/100mg	28.21	22.57	33.85	Gamma	Local charge
Bevacizumab/100mg	156.90	141.63	212.87	Gamma	Local charge
Topotecan/1mg	7.28	5.82	8.74	Gamma	Local charge
Cost of serious AEs					
Anemia	138.57	110.86	531.70	Gamma	(15, 19)
White blood cell count decreased	473.01	378.41	567.61	Gamma	(21)
Neutrophil count decreased	399.63	319.70	479.56	Gamma	(19)
Platelet count decreased	1,094.70	875.76	3,605.13	Gamma	(19, 22)
Urinary tract infection	126.03	100.82	151.24	Gamma	(19)
Hypokalemia	3,271.49	2,617.19	3,925.79	Gamma	(23)
Utility					
PFS	0.76	0.61	0.91	Beta	(19)
PD	0.52	0.42	0.62	Beta	(19)
Disutility due to grade ≥E;3 AEs	0.28	0.22	0.34	Beta	(6)
Risk of serious AEs in cadonilimab plus chemotherapy group					
Anemia	0.17	0.14	0.20	Beta	(13)
White blood cell count decreased	0.28	0.22	0.34	Beta	(13)
Neutrophil count decreased	0.41	0.33	0.49	Beta	(13)
Platelet count decreased	0.14	0.11	0.17	Beta	(13)
Urinary tract infection	0.06	0.05	0.07	Beta	(13)
Hypokalemia	0.06	0.05	0.07	Beta	(13)
Risk of serious AEs in chemotherapy group					
Anemia	0.26	0.21	0.31	Beta	(13)
White blood cell count decreased	0.36	0.29	0.43	Beta	(13)
Neutrophil count decreased	0.46	0.37	0.55	Beta	(13)
Platelet count decreased	0.12	0.10	0.14	Beta	(13)
Urinary tract infection	0.05	0.04	0.06	Beta	(13)
Hypokalemia	0.05	0.04	0.06	Beta	(13)
Body surface area (m^2^)	1.64	1.31	1.97	Normal	(15)
Body weight (kg)	60	48	72	Normal	(15)
Creatinine clearance (mL/min)	70	56	84	Normal	(15)
AUC	4.5	4	5	Normal	(13)
Discount rate (%)	5	0	8	Fixed	CGPE

OS, overall survival; PFS, progression-free survival; CT, computed tomography; AE, adverse event.

### 2.2 Model overview

**Figure 1:**
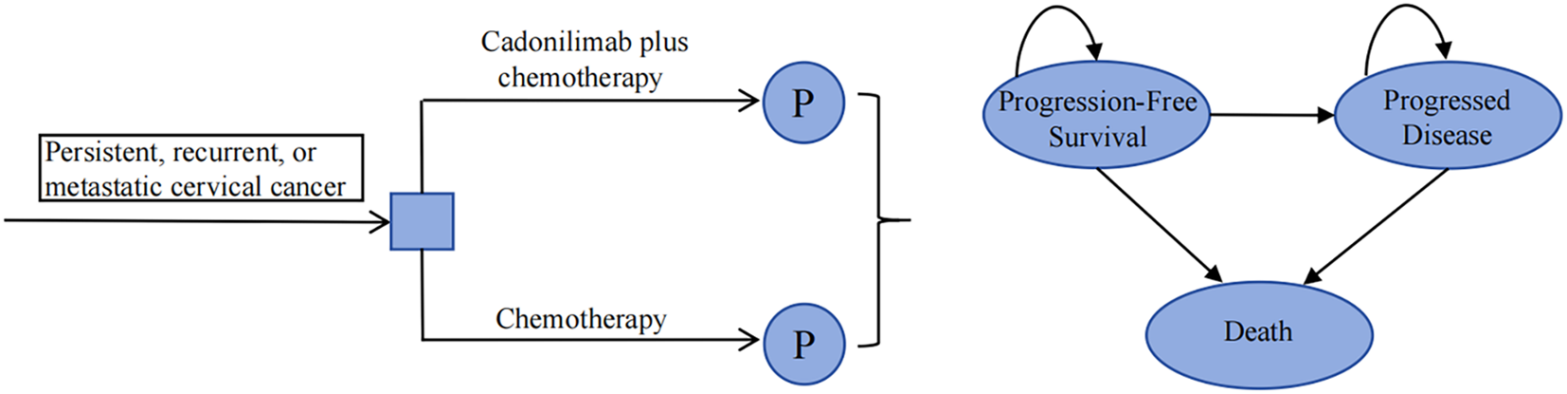
Model structure. P, partitioned survival model.

A partitioned survival model was developed using TreeAge Pro 2022 to simulate disease progression (**Figure 1**). The model comprised three mutually exclusive health states: PFS, progressed disease (PD), and death. The proportion of patients in the PFS state was determined by the area under the PFS curve, while the proportion of patients in the PD state was calculated based on the disparity in area between the OS curve and the PFS curve. The model utilized a cycle length of 3 weeks, aligning with the dosing schedule of the clinical trial. The time horizon was set at 10 years due to the poor prognosis, as the 5-year OS rate is only 15% in China (16). The primary outcomes included total costs, quality-adjusted life-years (QALYs), and incremental cost-effectiveness ratio (ICER). If the ICER is less than the predetermined willingness-to-pay (WTP) threshold, the intervention is considered cost-effective; otherwise, it is not. Following the 2020 China guidelines for pharmacoeconomic evaluations (CGPE) and the World Health Organization’s recommendation (17), the WTP threshold was established at three times China’s per capita gross domestic product (GDP) in 2023, amounting to $38,042/QALY. Moreover, incremental net monetary benefit (INMB) and incremental net health benefit (INHB) were employed as additional outcomes. A value greater than 0 indicates that the intervention is economically favorable.

Data points were extracted from the OS and PFS curves reported in the COMPASSION-16 trial using the WebPlotDigitizer software. Subsequently, individual patient data were reconstructed according to the algorithm developed by Guyot et al. (18). Standard survival parameter models, including Exponential, Weibull, Log-normal, Log-logistic, Gompertz, and Generalized gamma, were applied to fit and extrapolate the survival curves beyond the clinical trial follow-up period. The optimal distribution was selected based on the Akaike Information Criterion (AIC) and Bayesian Information Criterion (BIC), in combination with visual inspection (**Supplementary Figure 1**, **Supplementary Table 3**). The model accuracy was validated internally by comparing the reconstructed survival curves with the COMPASSION-16 trial data and externally by comparing the model with survival data from the KEYNOTE-826 and BEATcc trials. The key parameters for the optimal distributions of each survival curve were outlined in **Table 1**.

### 2.3 Cost and utility input

From a healthcare system perspective, this study only took into account direct medical expenses, as shown in **Table 1**. According to the COMPASSION-16 trial, routine laboratory tests, including 12-lead electrocardiogram, hematology, blood chemistry, and urinalysis, were conducted during each cycle. Coagulation tests and thyroid tests were performed every two cycles. Contrast-enhanced computed tomography (CT) scans were carried out every 6 weeks for the first 24 weeks, every 9 weeks for weeks 25-51, and then every 12 weeks until disease progression. Other expenses included drug costs, best supportive care, terminal care, drug administration, and management of adverse events (AEs). Drug prices were sourced from the Hunan Province Medical Security Bureau (https://healthcare.hnybj.com.cn) database, reflecting national public hospital pricing under drug price linkage policy in China. However, when applying the model to other countries, it is important to consider updated cost information, including drug prices. Paclitaxel was included in the national volume-based procurement program, with its price set by the winning bid, while the prices of other drugs were calculated as the average from all available manufacturers. Other cost values were collected from the literature. Only grade ≥3 AEs with a risk ≥5% in any group were included in this study. It was assumed that all AEs occurred during the first cycle (24). In addition, based on expert opinions and literature, it was assumed that a second-line treatment after disease progression consisted of topotecan (0.75 mg/m^2^, day 1-3, every 3 weeks) and paclitaxel (175 mg/m², day 1, every 3 weeks) (25). All expenses were adjusted to the 2023 USD using the Consumer Price Index and the 2023 average exchange rate (1 USD = 7.0467 RMB). Since the quality-of-life data were not reported in the COMPASSION-16 trial, utility values and AE-related disutility values were obtained from published literature, **Table 1**. Annual discounting at 5% was applied to both costs and utilities, as per the 2020 version of the CGPE.

### 2.4 Sensitivity analyses

Sensitivity analyses, including one-way and probabilistic sensitivity analyses (PSA), were conducted to address model uncertainty. One-way sensitivity analysis was used to assess the effect of individual parameter changes on the model outcomes, with the ICER serving as the primary indicator. The price ranges for drugs with multiple manufacturers in the database were established based on the minimum and maximum values. For drugs produced by a single manufacturer and other key parameters, the ranges were set at ±20% of the baseline value. However, due to noticeable variations in reported management costs related to anaemia and decreased platelet counts across the literature, the maximum values were extended to encompass these reported figures. A PSA was conducted to assess the impacts of concurrent variations in all parameters on the outcomes. This approach involved sampling parameters from predetermined distributions, and performing 1000 iterations of Monte Carlo simulation. The distributions of key parameters were detailed in **Table 1**.

### 2.5 Scenario analyses

#### 2.5.1 Scenario 1

To assess the impact of employing different distributions for extrapolating survival data beyond the observation period of the COMPASSION-16 trial, each survival curve was fitted with a suboptimal distribution exhibiting AIC and BIC values slightly higher than the optimal distribution in scenario 1. The key parameters were shown in **Supplementary Table 4**.

#### 2.5.2 Scenario 2

The model utilized utility values from the literature instead of directly from the COMPASSION-16 trial data. To test the robustness of the model results, an alternative set of utility values was applied in scenario 2, including a PFS utility value of 0.827, a PD utility value of 0.779, and a disutility value of 0.28 for severe AEs (6, 26).

### 2.6 Subgroup analyses

In order to assess the cost-effectiveness of cadonilimab in patients with various characteristics, comprehensive subgroup analyses were performed based on the COMPASSION-16 trial. The subgroup analysis included common prognostic factors for cancer patients, such as age, pathological diagnosis, metastatic status, and physical condition at baseline. Additionally, PD-L1 expression levels, which can impact the outcomes of immunotherapy, were taken into account, along with variations in treatment regimens, such as the inclusion of bevacizumab and the use of different platinum-based drugs. In the absence of survival data for each subgroup, we applied a method that involved adjusting HRs for OS and PFS, under the assumption of proportional hazards (19).

## 3 Results

### 3.1 Base-case analysis

The results of the base-case analysis were shown in **Table 2**. The cadonilimab plus chemotherapy group gained an extra 0.61 QALYs over the chemotherapy group at an additional cost of $42,486.54, leading to an ICER of $70,220.88/QALY. This exceeded the WTP threshold, indicating that cadonilimab combination treatment was not cost-effective. Furthermore, the INMB was -$19,469.55, and the INHB was -0.51 QALYs, which were consistent with the ICER results. To reduce the ICER to the WTP threshold of $38,042/QALY, the price of cadonilimab would need to decrease by an additional 77.47%.

**Table 2:** Results of the base-case and scenario analyses.

Treatment	Total cost ($)	Incremental costs ($)	QALYs	Incremental QALYs	ICER ($/QALY)	INMB ($)	INHB (QALYs)
Base-case analysis							
Chemotherapy	25,707.10	–	1.12	–	–	–	–
Cadonilimab plus chemotherapy	68,193.65	42,486.54	1.73	0.61	70,220.88	-19,469.55	-0.51
Scenario analysis 1							
Chemotherapy	23,628.96	–	0.98	–	–	–	–
Cadonilimab plus chemotherapy	65,690.69	42,061.73	1.49	0.51	82,056.94	-22,561.70	-0.59
Scenario analysis 2							
Chemotherapy	25,707.10	–	1.54	–	–	–	–
Cadonilimab plus chemotherapy	68,193.65	42,486.54	2.27	0.73	58,588.01	-14,899.45	-0.39

QALYs, quality-adjusted life-years; ICER, incremental cost-effectiveness ratio; INMB, incremental net monetary benefit; INHB, incremental net health benefit.

### 3.2 Sensitivity analyses

**Figure 2:**
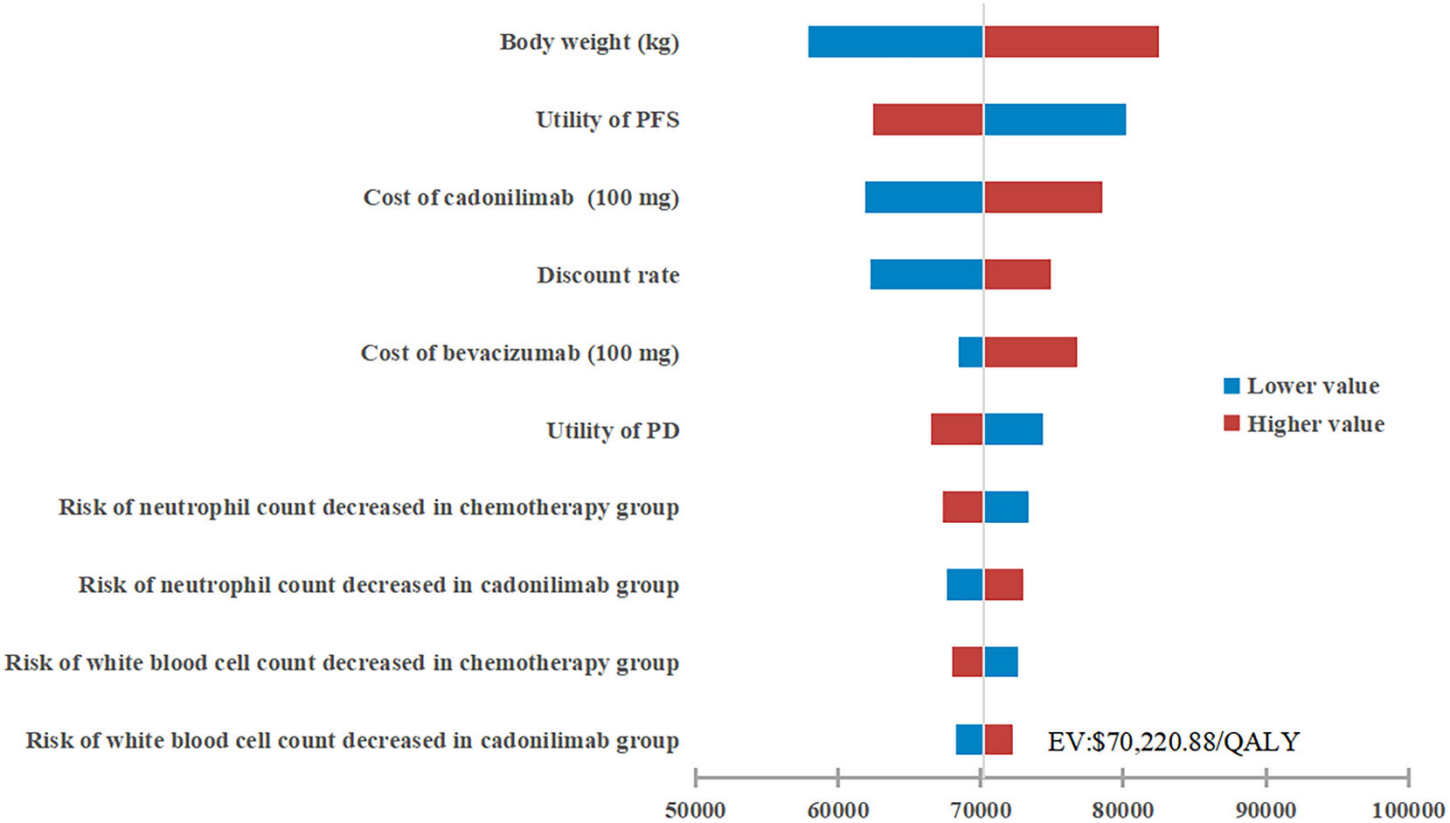
One-way sensitivity analysis. PFS, progression-free survival; PD, progressed disease; QALY, quality-adjusted life-year; ICER, incremental cost-effectiveness ratio.

**Figure 3:**
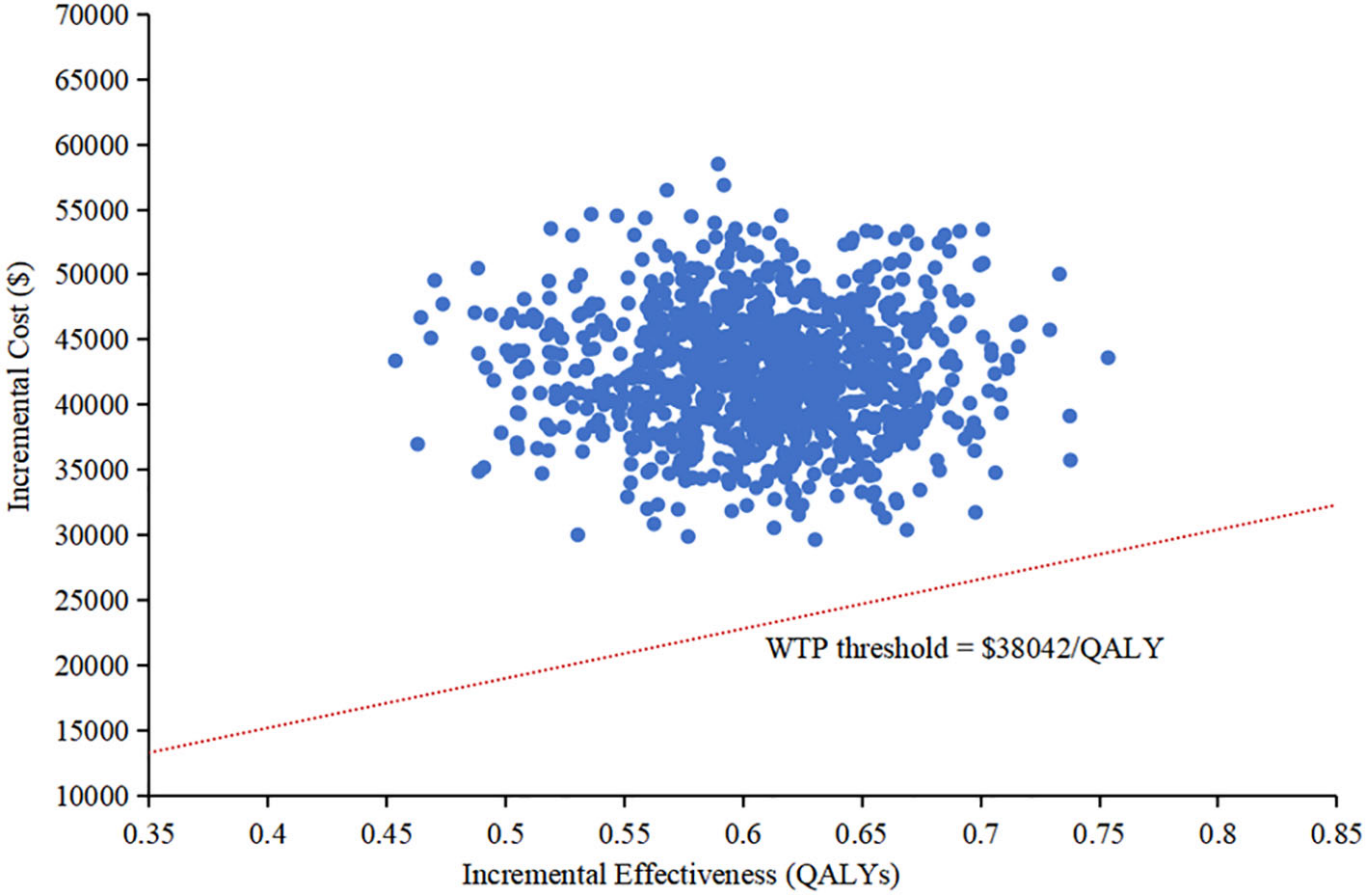
Cost-effectiveness acceptability curve. QALY, quality-adjusted life-year; WTP, willingness-to-pay.

**Figure 4:**
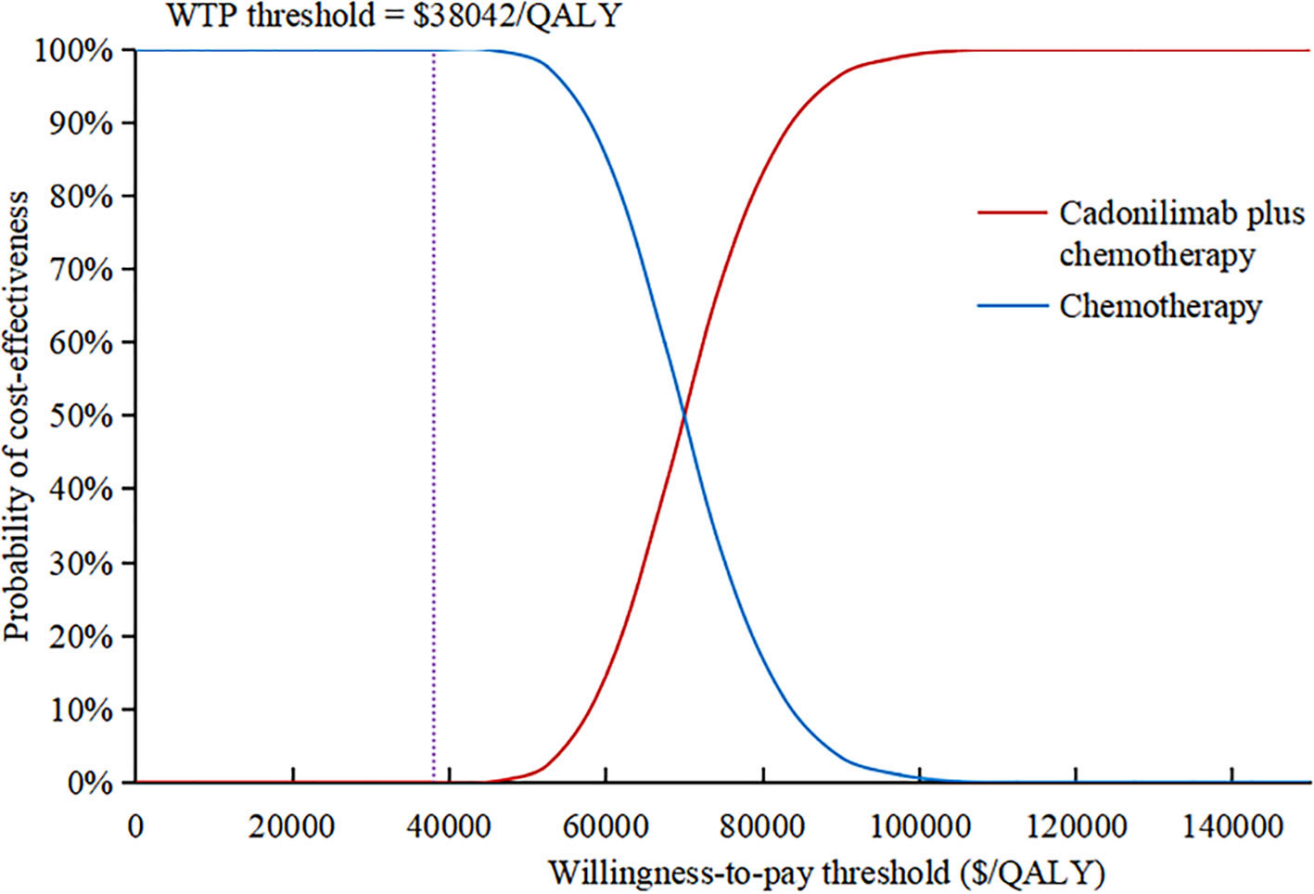
Scatter plot in the probabilistic sensitivity analysis. QALY, quality-adjusted life-year; WTP, willingness-to-pay.

The results of the one-way sensitivity analysis were presented in the tornado diagram, as shown in **Figure 2**. Body weight, PFS utility, and the cost of cadonilimab were identified as the three most influential parameters affecting the results. The model results remained stable as each parameter varied within the predetermined range, indicating the robustness of the model. The results of the PSA were depicted in the cost-effectiveness acceptability curve (**Figure 3**). At a WTP threshold of $38,042/QALY, the probability of cadonilimab plus chemotherapy being cost-effective was 0%. A scatter plot illustrating this was presented in **Figure 4**.

### 3.3 Scenario analyses

The ICER values were $82,056.94/QALY in scenario analysis 1 and $58,588.01/QALY in scenario analysis 2, both exceeding the predefined WTP threshold. Both INMB and INHB values were negative and consistent with those of the base-case analysis. Details were summarized in **Table 2**.

### 3.4 Subgroup analyses

The subgroup analyses revealed that cadonilimab plus chemotherapy was not cost-effective for most patients, aligning with the base-case findings, except for those who did not receive bevacizumab (**Table 3**). In this specific subgroup, the ICER value was $33,829.84/QALY, with an INMB of $4,606.01 and an INHB of 0.12 QALYs, indicating that cadonilimab plus chemotherapy was cost-effective; the likelihood of cadonilimab combination treatment being cost-effective was 80.80% at a WTP threshold of $38,042/QALY. Additionally, cadonilimab plus chemotherapy did not achieve cost-effectiveness in patients with any level of PD-L1 expression, and economic outcomes were not proportional to PD-L1 expression levels.

**Table 3:** Results of the subgroup analyses.

Subgroup	HR for OS (95% CI)	HR for PFS (95% CI)	ICER ($/QALY)	INMB ($)	INHB (QALYs)	Cost-effective probability of cadonilimab plus chemotherapy
Age						
Age < 65	0.69 (0.50-0.95)	0.7 (0.53-0.92)	77,684.77	-21,521.09	-0.57	0%
Age ≥ 65	0.49 (0.27-0.91)	0.40 (0.22-0.71)	52,010.22	-16,508.58	-0.43	1.40%
ECOG performance status score						
0	0.79 (0.46-1.36)	0.64 (0.41-1.00)	100,789.45	-26,262.50	-0.69	0%
1	0.57 (0.41-0.79)	0.61 (0.46-0.82)	59,896.11	-17,891.09	-0.47	0%
Concomitant bevacizumab						
Yes	0.84 (0.56-1.26)	0.81 (0.58-1.13)	125,518.84	-24,564.98	-0.65	0%
No	0.50 (0.33-0.75)	0.46 (0.32-0.66)	33,829.84	4,606.01	0.12	80.80%
Previous concurrent chemoradiotherapy						
Yes	0.54 (0.35-0.82)	0.55 (0.39-0.79)	56,913.27	-17,433.65	-0.46	0%
No	0.76 (0.52-1.12)	0.72 (0.52-1.01)	93,954.83	-23,706.66	-0.62	0%
Pathological diagnosis						
Squamous cell carcinoma	0.64 (0.47-0.88)	0.58 (0.44-0.76)	69,387.96	-21,772.69	-0.57	0%
Non-Squamous cell carcinoma	0.63 (0.33-1.22)	0.94 (0.52-1.69)	63,839.89	-14,693.84	-0.39	0.10%
Metastatic						
Yes	0.73 (0.52-1.02)	0.71 (0.53-0.94)	86,265.69	-22,840.70	-0.60	0%
No	0.48 (0.27-0.86)	0.46 (0.28-0.78)	51,414.70	-15,330.89	-0.40	1.20%
PD-L1 combined positive score						
<1	0.77 (0.44-1.34)	0.73 (0.46-1.17)	75,525.59	-20,531.00	-0.54	0%
≥1	0.69 (0.49-0.97)	0.62(0.46-0.83)	77,677.00	-23,080.67	-0.61	0%
≥10	0.68 (0.42-1.08)	0.53(0.35-0.79)	75,166.41	-24,358.70	-0.64	0%
Cisplatin or carboplatin						
Cisplatin	0.43 (0.27-0.70)	0.54(0.37-0.80)	46,643.61	-10,550.72	-0.28	5.40%
Carboplatin	0.82 (0.57-1.18)	0.71(0.52-0.97)	113,291.43	-25,953.58	-0.68	0%

HR, hazard ratio; CI, confidence interval; OS, overall survival; PFS, progression-free survival; QALYs, quality-adjusted life-years; ICER, incremental cost-effectiveness ratio; INMB, incremental net monetary benefit; INHB, incremental net health benefit; ECOG, Eastern Cooperative Oncology Group; PD-L1, programmed death ligand 1.

## 4 Discussion

In the therapeutic management of CC, combination therapy involving two immune checkpoint inhibitors (ICIs) that target PD-1 and CTLA-4 separately has demonstrated synergistic effects (27, 28). However, this therapeutic strategy is associated with higher rates of AEs, thus constraining its clinical adoption (29–31). Comparatively, the PD-1 and CTLA-4 bispecific antibody demonstrates superior antitumor efficacy with manageable safety profiles (32). Cadonilimab, the first approved PD-1/CTLA-4 blocker, has been authorized for the treatment of cervical and gastric cancers in China.

Although cadonilimab has been included in China’s Basic Medical Insurance Drug List following successful price negotiation, our pharmacoeconomic analysis revealed cadonilimab combination treatment failed to demonstrate cost-effectiveness as first-line therapy for p/r/m CC compared to standard chemotherapy. The ICER value was $70,220.88/QALY, exceeding the WTP threshold. Despite this, cadonilimab still holds a significant price advantage compared to other ICIs that have shown positive results in phase III clinical trials, such as pembrolizumab and atezolizumab. Owing to their high pricing, pembrolizumab and atezolizumab did not achieve cost-effectiveness in China across all analyzed subgroups (15, 19, 22). Similarly, atezolizumab failed to demonstrate cost-effectiveness in the United States (6, 33). However, pembrolizumab combination treatment in PD-L1 positive populations might be cost-effective in the United States (26, 34). Although cadonilimab has not yet been launched in other countries, its cost-effectiveness may be promising in developed countries due to a higher WTP threshold and its price advantage compared to other ICIs.

The one-way sensitivity analysis identified body weight, PFS utility, and the cost of cadonilimab as the most influential factors affecting the model outcomes. Specifically, body weight affected the dosages of cadonilimab and bevacizumab, which indirectly highlighting the impact of drug costs. To make the cadonilimab combination treatment cost-effective, a further price reduction of 77.47% for cadonilimab would be required. However, achieving such a substantial reduction in the short term remains challenging, as drugs added to the Basic Medical Insurance Drug List via negotiations face annual price cuts ranging from 0-25% over the first four years in China. Subgroup analyses demonstrated cost-effectiveness for cadonilimab plus chemotherapy in patients who were not treated with bevacizumab. This might be largely due to the reduced drug costs resulting from the exclusion of bevacizumab, and the contribution of cadonilimab to survival benefits appeared to outweigh that of bevacizumab (35). When managing CC, there seems to be a cumulative effect in patients’ responses to ICIs, bevacizumab, and chemotherapy. However, not all patients are suitable candidates for bevacizumab because of the increased risk of fistula formation or bowel perforation associated with anti-angiogenic monoclonal antibodies (36). The study concluded that cadonilimab combination treatment was cost-effective for this patient subset. Furthermore, subgroup analyses revealed that the economic outcomes of cadonilimab plus chemotherapy were not significantly correlated with PD-L1 expression levels, distinguishing it from other ICIs like pembrolizumab, which showed a decreased probability of cost-effectiveness with lower PD-L1 expression levels (19). This finding aligned with the remarkable clinical benefits observed in PD-L1-negative patients treated with cadonilimab, underscoring its substantial advantage over other ICIs.

This study has several limitations. First, the COMPASSION-16 trial was conducted in China, which helped reduce bias stemming from regional heterogeneity. However, when applying this model to regions outside of China, it would be necessary to collect additional data for validation. Second, in the COMPASSION-16 trial, quality-of-life data were not reported; therefore, the utility values used in this study were obtained from the literature, which may differ from real-world data. However, the one-way sensitivity analysis and scenario analysis 2 confirmed the robustness of the model. Third, this study only took into account the costs and utility values of AEs of grade 3 or higher and did not consider the long-term impact of AEs due to data limitations. Although this constraint might affect the accuracy of the model, the tornado diagram indicated that AE-related parameters had a limited impact on the primary outcomes. Fourth, survival curve extrapolation using standard parametric models introduces inherent uncertainties to the model, compounded by immature OS data from COMPASSION-16. Therefore, it is crucial to validate the model with long-term real-world data in the future, despite confirming that the results align with the base-case analysis using suboptimal distributions in scenario analysis 1. Finally, income inequality between regions may impact the conclusions of this study. For example, it may lead to an underestimation of the cost-effectiveness of cadonilimab in regions with higher per capita GDP.

In conclusion, for patients with p/r/m CC, cadonilimab plus chemotherapy is not cost-effective compared to standard chemotherapy as a first-line treatment. However, it serves as a cost-effective alternative for patients who are ineligible for bevacizumab. Designing clinical trials that focus on populations more likely to benefit significantly from this treatment, such as those for whom the use of bevacizumab is not feasible, will enhance the understanding of the efficacy and cost-effectiveness of cadonilimab in the future.

## Data Availability

The original contributions presented in the study are included in the article/**Supplementary Material**. Further inquiries can be directed to the corresponding author.
