# Supplementary material for: Cadonilimab plus chemotherapy as first-line treatment for persistent, recurrent, or metastatic cervical cancer: a cost-effectiveness analysis
Source: Front Immunol. 2025 Apr 3;16:1562875. doi: 10.3389/fimmu.2025.1562875 (PMC12003394; doi:10.3389/fimmu.2025.1562875)
Supplement: Supplementary file 1 [file DataSheet1.pdf]

Supplementary Material

1 Supplementary Tables

Supplementary Table 1. CHEERS Checklist (2022)

Topic	No.	Item	Reported?
**Title and abstract**			
Title	1	Identify the study as an economic evaluation and specify the interventions being compared.	Yes
Abstract	2	Provide a structured summary that highlights context, key methods, results, and alternative analyses.	Yes
**Introduction**			
Background and objectives	3	Give the context for the study, the study question, and its practical relevance for decision making in policy or practice.	Yes
**Methods**			
Health economic analysis plan	4	Indicate whether a health economic analysis plan was developed and where available.	Yes
Study population	5	Describe characteristics of the study population (such as age range, demographics, socioeconomic, or clinical characteristics).	Yes
Setting and location	6	Provide relevant contextual information that may influence findings.	Yes
Comparators	7	Describe the interventions or strategies being compared and why chosen.	Yes
Perspective	8	State the perspective(s) adopted by the study and why chosen.	Yes
Time horizon	9	State the time horizon for the study and why appropriate.	Yes
Discount rate	10	Report the discount rate(s) and reason chosen.	Yes
Selection of outcomes	11	Describe what outcomes were used as the measure(s) of benefit(s) and harm(s).	Yes
Measurement of outcomes	12	Describe how outcomes used to capture benefit(s) and harm(s) were measured.	Yes
Valuation of outcomes	13	Describe the population and methods used to measure and value outcomes.	Yes
Measurement and valuation of resources and costs	14	Describe how costs were valued.	Yes
Currency, price date, and conversion	15	Report the dates of the estimated resource quantities and unit costs, plus the currency and year of conversion.	Yes
Rationale and description of model	16	If modelling is used, describe in detail and why used. Report if the model is publicly available and where it can be accessed.	Yes

Topic	No.	Item	Reported?
Analytics and assumptions	17	Describe any methods for analysing or statistically transforming data, any extrapolation methods, and approaches for validating any model used.	Yes
Characterising heterogeneity	18	Describe any methods used for estimating how the results of the study vary for subgroups.	Yes
Characterising distributional effects	19	Describe how impacts are distributed across different individuals or adjustments made to reflect priority populations.	Yes
Characterising uncertainty	20	Describe methods to characterise any sources of uncertainty in the analysis.	Yes
Approach to engagement with patients and others affected by the study	21	Describe any approaches to engage patients or service recipients, the general public, communities, or stakeholders (such as clinicians or payers) in the design of the study.	Not applicable
**Results**			
Study parameters	22	Report all analytic inputs (such as values, ranges, references) including uncertainty or distributional assumptions.	Yes
Summary of main results	23	Report the mean values for the main categories of costs and outcomes of interest and summarise them in the most appropriate overall measure.	Yes
Effect of uncertainty	24	Describe how uncertainty about analytic judgments, inputs, or projections affect findings. Report the effect of choice of discount rate and time horizon, if applicable.	Yes
Effect of engagement with patients and others affected by the study	25	Report on any difference patient/service recipient, general public, community, or stakeholder involvement made to the approach or findings of the study	Not applicable
**Discussion**			
Study findings, limitations, generalisability, and current knowledge	26	Report key findings, limitations, ethical or equity considerations not captured, and how these could affect patients, policy, or practice.	Yes
Other relevant information			
Source of funding	27	Describe how the study was funded and any role of the funder in the identification, design, conduct, and reporting of the analysis	Yes
Conflicts of interest	28	Report authors conflicts of interest according to journal or International Committee of Medical Journal Editors requirements.	Yes

Supplementary Table 2. Specific first-line treatment protocols

Drug	Cadonilimab plus chemotherapy group		Chemotherapy group	
	Usage and dosage	Rate	Usage and dosage	Rate
Cadonilimab	10 mg/kg was administered intravenously every 3 weeks for 2 years	1	-	-
Cisplatin	50 mg/m² was administered intravenously every 3 weeks for 6 cycles	0.41	50 mg/m² was administered intravenously every 3 weeks for 6 cycles	0.45
Carboplatin	area under the curve 4.5 was administered intravenously every 3 weeks for 6 cycles	0.59	area under the curve 4.5 was administered intravenously every 3 weeks for 6 cycles	0.55
Paclitaxel	175 mg/m² was administered intravenously every 3 weeks for 6 cycles	1	175 mg/m² was administered intravenously every 3 weeks for 6 cycles	1
Bevacizumab	15 mg/kg was administered intravenously every 3 weeks for 2 years	0.6	15 mg/kg was administered intravenously every 3 weeks for 2 years	0.59

Supplementary Table 3. Summary of the statistical goodness-of-fit of KM curve.

	Exponential	Weibull	Gamma	Generalized gamma	Gompertz	Log-normal	Log-logistic
OS curve							
Cadonilimab plus chemotherapy							
AIC	836.7832	826.4496	826.2448	828.2417	830.3894	831.1456	825.7580
BIC	840.1859	833.2549	833.0502	838.4497	837.1947	837.9510	832.5634
Chemotherapy							
AIC	972.6002	954.2626	951.3368	951.3139	965.5757	951.3186	948.2766
BIC	976.0073	961.0769	958.1511	961.5354	972.3900	958.1329	955.0909
PFS curve							
Cadonilimab plus chemotherapy							
AIC	892.3545	871.2356	868.6060	869.2800	882.9203	869.0824	865.8905
BIC	895.7572	878.0410	875.4113	879.4880	889.7257	875.8878	872.6959
Chemotherapy							
AIC	1027.6929	994.7706	986.6110	979.3352	1017.4128	977.5548	977.1933
BIC	1031.1000	1001.5850	993.4253	989.5567	1024.2272	984.3691	984.0076

Supplementary Table 4. The key parameters for the suboptimal distributions of each survival curve.

Survival curve	Suboptimal distribution	Key parameters
OS curve of cadonilimab plus chemotherapy arm	Gamma	shape=1.6406, rate=0.0412
OS curve of chemotherapy arm	Gamma	shape=1.8636, rate=0.0670
PFS curve of cadonilimab plus chemotherapy arm	Gamma	shape=1.9105, rate=0.1149
PFS curve of chemotherapy arm	Log-normal	meanlog=2.2592, sdlog=0.8025

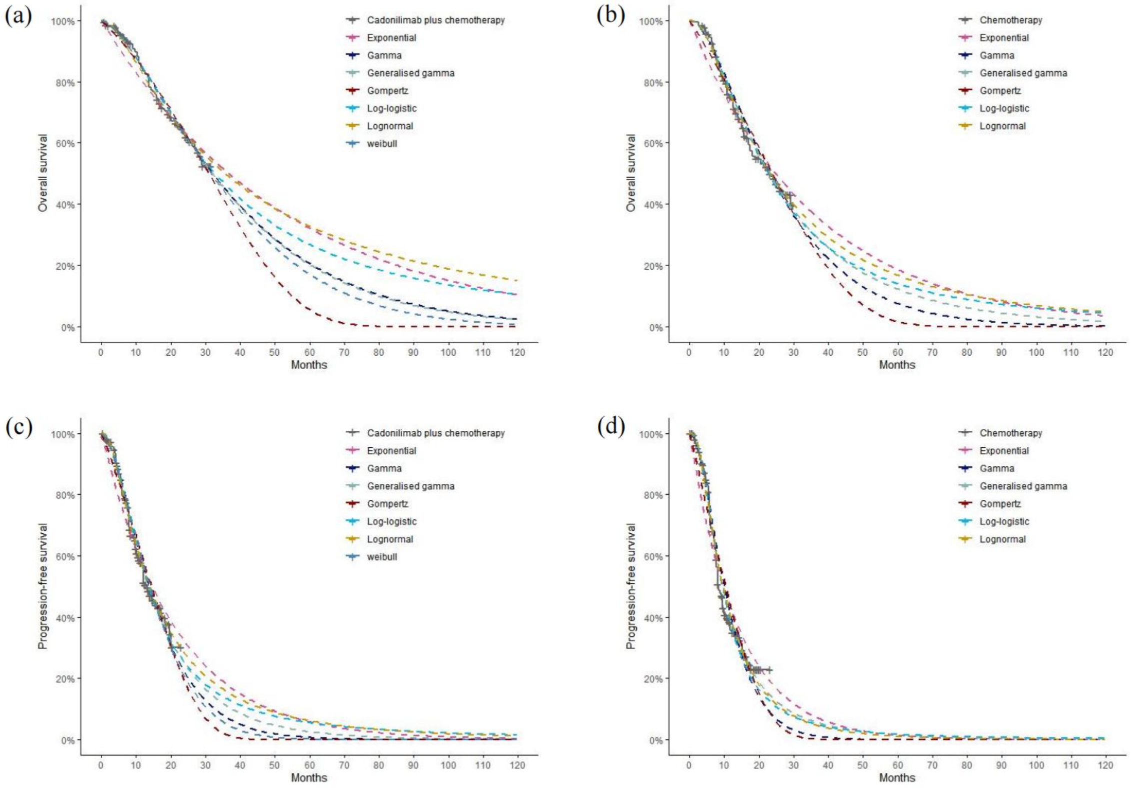

Supplementary Figure 1. Fitting and extrapolation of Kaplan Meier survival curve. (a) The results of OS curve in cadonilimab plus chemotherapy arm. (b) The results of OS curve in chemotherapy arm. (c) The results of PFS curve in cadonilimab plus chemotherapy arm. (b) The results of PFS curve in chemotherapy arm. OS, overall survival; PFS, progression-free survival.
